# Supplementary material for: Rapid computation of high-level visual surprise
Source: iScience. 2025 Nov 19;28(12):114121. doi: 10.1016/j.isci.2025.114121 (PMC12719766; doi:10.1016/j.isci.2025.114121)
Supplement: Document S1. Figure S1 and Tables S1–S2 [file mmc1.pdf]

iScience, Volume *28*

Supplemental information

Rapid computation of high-level visual surprise

David Richter, Paula Pena, and María Ruz

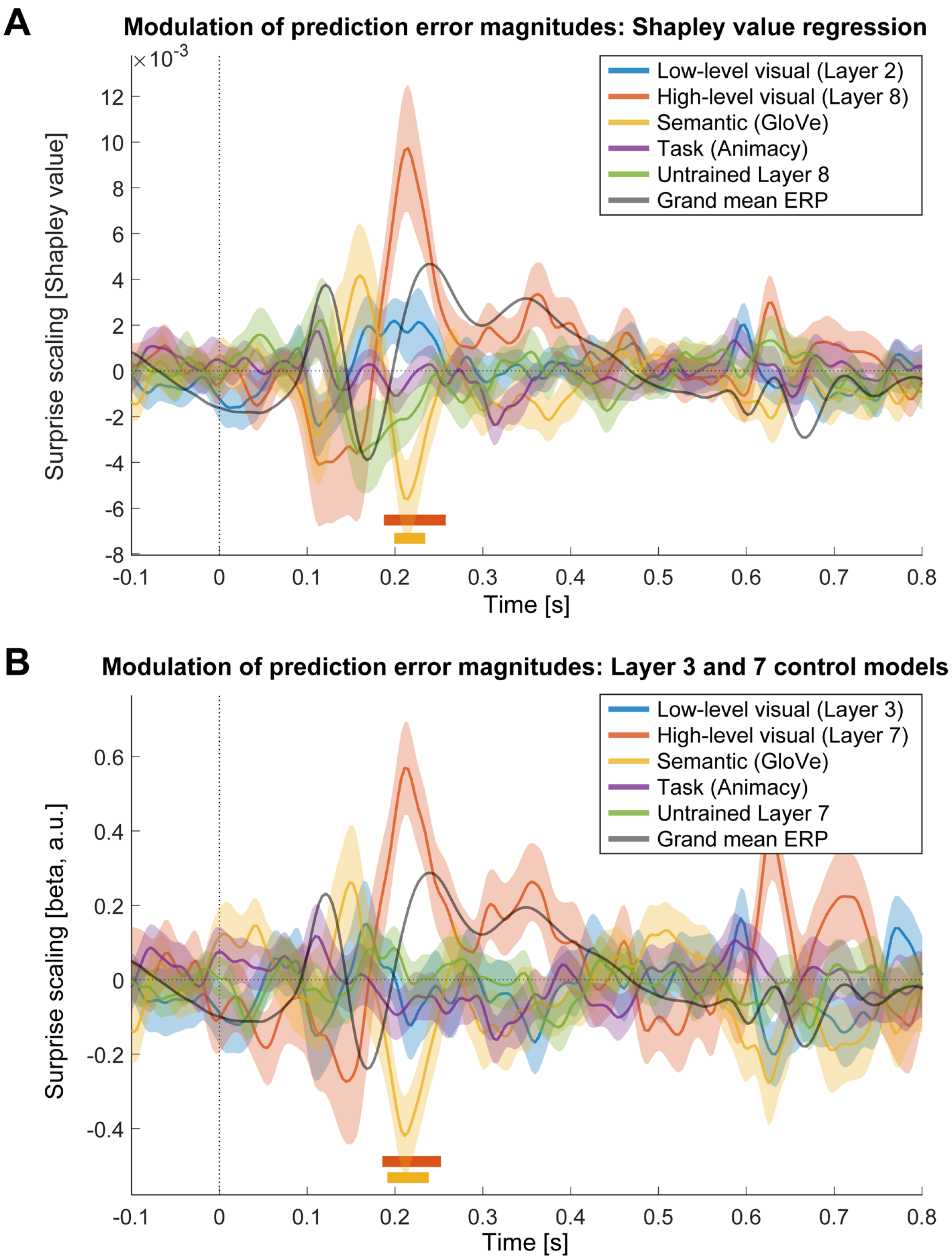

Figure S1. Control analyses regressing model-based surprise onto evoked EEG activity. A) Control analysis using Shapley regression. To address potential concerns arising from correlation between predictors, we repeated the surprise scaling analysis using Shapley value regression. In brief, it computes the average marginal contribution over all subsets of models for the five predictors, thereby helping to address possible issues arising from multicollinearity. While Shapley values are unsigned, we reconstructed signs from the original coefficients. Results replicated those reported in our primary analysis using ordinary least squares regression, showing a pronounced positive modulation of neural responses by high-level visual surprise and a slight negative modulation by semantic surprise. Low-level visual surprise again did not scale responses. **B)** Control analysis using neighboring layers of the primary surprise scaling analysis. To ensure that results were not contingent on the (a priori) selected DNN layers, we repeated the surprise scaling analysis using neighboring layers. Thus, in this analysis layer 3 was used as low-

level and layer 7 as high-level visual surprise model. Note, in our implementation of AlexNet layer 8 is the final layer before softmax, thus the neighboring layer for layer 8 containing high-level visual representations is layer 7. Results replicated the primary findings, showing a clear upregulation of visually evoked responses by high-level visual surprise approximately 200 ms after stimulus onset. As in the main analysis, we again found no modulation of ERP amplitudes by low-level visual surprise (here layer 3). Therefore, this control analysis demonstrates that our results are not contingent on the precise layers selected for analysis but replicate across neighboring layers, reinforcing our conclusion that visually evoked responses are primarily modulated by high-level visual surprise. Colored bars above the abscissa denote statistically significant clusters ($p_{cluster} < 0.05$).

ERP	Contrast	Test statistic: $t_{(37)}$	P value	Effect size: d_z
P2	Layer 8 vs Layer 2	3.79	0.002	0.61
P2	Layer 8 vs GloVe	5.56	2.5e-6	0.90
P2	Layer 8 vs Animacy	3.72	0.001	0.60
P2	Layer 8 vs Control model	5.43	3.8e-6	0.88

Table S1. Results of paired t-tests contrasting the modulation ERP magnitudes by high-level visual surprise (layer 8) against the four other surprise models. ERP time windows were defined per participant using the maximal positive deflection within commonly reported time windows for the P2 potential. Beta coefficients were averaged per participant within these time windows and contrasted between models on the group level. Test statistics reflect paired t-tests. P values are FDR corrected using the Benjamini and Hochberg method. Effect sizes are given as Cohen's d_z.

ERP	Surprise model	Test stat.: $t_{(37)}$	P value	Effect size: d_z	BF_{10}
P1	Low-level visual (layer 2)	-2.47	0.077	-0.40	2.473
P1	High-level visual (layer 8)	-0.63	0.703	-0.10	0.210
P1	Semantic (GloVe)	-0.76	0.669	-0.12	0.228
P1	Task (Animacy)	0.14	0.893	0.02	0.176
P1	Control (untrained layer 8)	0.65	0.703	0.10	0.212
N1	Low-level visual (layer 2)	1.13	0.478	0.18	0.314
N1	High-level visual (layer 8)	-1.57	0.314	-0.25	0.535
N1	Semantic (GloVe)	2.01	0.183	0.33	1.071
N1	Task (Animacy)	-0.35	0.823	-0.06	0.185
N1	Control (untrained layer 8)	-2.89	0.054	-0.47	6.019
P2	Low-level visual (layer 2)	0.31	0.823	0.05	0.183
**P2**	**High-level visual (layer 8)**	**5.46**	**8.4e-5**	**0.89**	**5578.153**
P2	Semantic (GloVe)	-2.85	0.054	-0.46	5.605
P2	Task (Animacy)	-0.89	0.617	-0.14	0.253
P2	Control (untrained layer 8)	-1.50	0.322	-0.24	0.489
N2	Low-level visual (layer 2)	1.37	0.373	0.22	0.413
N2	High-level visual (layer 8)	2.68	0.054	0.44	3.883
N2	Semantic (GloVe)	-1.94	0.189	-0.31	0.938
N2	Task (Animacy)	0.26	0.827	0.04	0.180
N2	Control (untrained layer 8)	-1.22	0.446	-0.20	0.345
P3	Low-level visual (layer 2)	0.39	0.823	0.06	0.188
P3	High-level visual (layer 8)	2.77	0.054	0.45	4.692
P3	Semantic (GloVe)	-1.70	0.273	-0.28	0.643
P3	Task (Animacy)	-0.86	0.617	-0.14	0.246
P3	Control (untrained layer 8)	0.38	0.823	0.06	0.187

Table S2. Results of one-sample t-tests contrasting the modulation of ERP magnitudes by surprise against zero (no modulation). ERP time windows were defined per participant using the maximal negative (N1, N2) or positive (P1, P2, P3) deflection within commonly reported time windows of the respective ERP. Beta coefficients were averaged per participant within these time windows and contrasted against zero at the group level. Test statistics reflect one-sample t-tests. P values are FDR corrected using the Benjamini and Hochberg method. Effect sizes are given as Cohen's d_z. Bayes factors are reported as BF10. $p_{corrected} < 0.05$ are marked in bold.
